# The Effectiveness of Intervention Programs for Perpetrators of Intimate Partner Violence with Substance Abuse and/or Mental Disorders: A Systematic Review

**DOI:** 10.1177/15248380241270063

**Published:** 2024-08-24

**Authors:** Marta Sousa, Joana Andrade, Andreia de Castro Rodrigues, Sónia Caridade, Olga Cunha

**Affiliations:** 1 School of Psychology, Psychology Research Center, University of Minho, Braga, Portugal; 2 William James Center for Research, ISPA—Instituto Universitário, Lisbon, Portugal; 3 Digital Human-Environment Interaction Lab, Universidade Lusófona do Porto, Porto, Portugal

**Keywords:** intimate partner violence, perpetrators, alcohol and drugs, mental health, intervention program

## Abstract

Despite the high prevalence and severity of intimate partner violence (IPV) perpetration among men with mental health (MH) problems and substance use (SU), there is limited evidence on the most effective ways to reduce IPV within these groups. Hence, the present systematic review aims to evaluate the effectiveness of psychological interventions for male IPV perpetrators with MH issues and SU problems. Five databases (B-On, Pubmed PsycInfo, Science Direct, and Scopus) were searched for studies examining the effectiveness of IPV interventions. Twenty-three studies met the inclusion criteria, with 13 interventions described. Interventions were grouped into (1) specific interventions for SU among IPV perpetrators (*k* = 8), (2) nonspecific interventions for SU among IPV perpetrators (*K* = 3), and (3) specific interventions targeting MH among IPV perpetrators (*k* = 2). Cognitive behavioral therapy and motivational interviewing techniques were the most common approaches. Both specific and nonspecific programs addressing SU problems showed some positive effects on perpetrators’ behavior and attitudes. However, data from the two intervention programs focusing on MH showed reduced symptoms and re-assaults but without significant differences between the conditions. Despite methodological shortcomings in the studies, the specific and nonspecific interventions targeting SU and IPV show promise, which hinders drawing firmer conclusions. Nonetheless, further research is necessary to deepen our understanding of the MH impact interventions on IPV perpetrators.

Intimate Partner Violence (IPV) encompasses violence perpetrated by both current and former spouses and partners, resulting in physical, sexual, or psychological harm (World Health Organization [WHO], 2013). IPV includes physical aggression, sexual coercion, psychological abuse, and controlling behaviors (Centers for Disease Control and Prevention [CDCP], 2016). IPV poses a substantial public health concern, impacting millions of individuals annually (Sardinha et al., 2022), with detrimental psychological and physical effects in both the short and long term for victims and their families (Stubbs & Szoeke, 2022). Although men can be victims of IPV (e.g., Bates, 2020) and women can perpetrate IPV (e.g., Mackay et al., 2018), or both roles can be assumed by either gender (e.g., Machado et al., 2024), IPV is still mainly committed by men against women (WHO, 2024). Women experience this form of violence to a greater extent, with almost one in three women worldwide having been subjected to physical and/or sexual violence by an intimate partner at least once in their lifetime (WHO, 2021). Consequently, perpetrators’ intervention programs (PIPs) are crucial for addressing this complex phenomenon (Manita & Matias, 2016). These programs can be directed toward men, women, or both partners, although most programs still address male perpetrators (Cunha, Pedrosa, et al., 2024).

Over the years, PIPs differed in terms of theoretical frameworks to prevent and diminish the risk of IPV (Babcock et al., 2004). The early interventions were based on the Duluth model, which views IPV as a manifestation of patriarchy or male socialization (Pence & Paymar, 1993; Pender, 2012). According to this perspective, the perpetrator uses violence to maintain power and control over their partner (Babcock et al., 2004; Pender, 2012). Therefore, these early PIPs are based on a gender perspective with a psychoeducational approach aimed at modifying the attitudes of male perpetrators toward women (Butters et al., 2021; Cunha & Caridade, 2023; Dutton & Corvo, 2007; Gannon et al., 2019). Although the Duluth model adopts an educational format, it also includes cognitive-behavioral techniques (Bohall et al., 2016). Years later, Cognitive-Behavioral Therapy (CBT) emerged as an alternative to the Duluth model. CBT for perpetrators of IPV aims to transform hostile cognitive biases, work on affect dysregulation, and address skill-based deficiencies, including assertiveness, communication, and problem-solving (Cunha et al., 2022; Cunha & Caridade, 2023; Cunha & Gonçalves, 2014; Wexler, 2020). More recently, new approaches based on mindfulness principles, such as Acceptance and Commitment Therapy, have been applied to perpetrators of IPV (Cunha, Pereira, et al., 2024; Cunha & Caridade, 2023; Zarling et al., 2019).

Unconvincing results about the effectiveness of PIPs have driven these changes. Indeed, the effectiveness of PIPs for IPV perpetrators is yet to be established as controversial results have been shown in meta-analyses, literature reviews, and experimental studies (Arias et al., 2013; Karakurt et al., 2019; Nesset et al., 2019; Tarzia et al., 2020). Although some experimental studies show positive results in promoting change (e.g., Capinha et al., 2023; Cunha & Gonçalves, 2015; Cunha et al., 2023), meta-analyses and systematic reviews in this field have shown very small or slightly negative effects in violence reduction (e.g., Cheng et al., 2021; Karakurt et al., 2019; Tarzia et al., 2020; Wilson et al., 2021). However, several variables have contributed to the inconsistency of evidence about IPV interventions’ effectiveness, namely differences in settings, duration, intervention goals, dropout rates, individual differences, and underlying theoretical frameworks (e.g., Henwood et al., 2015; Pinto e Silva et al., 2023). Specifically, there are instances where individual differences are not considered in interventions, neglecting the fundamental principle of psychological intervention: one size does not fit all (Easton & Crane, 2016). Following this approach, adopting the risk-need-responsivity (RNR) model (Bonta & Andrews, 2016)—which postulates that intervention must consider the risk a perpetrator presents, their criminogenic needs, and their ability to respond—has shown positive results in the short-to-medium follow-up (Travers et al., 2021). Moreover, interventions must address the key risk factors of IPV, which include substance abuse, poor mental health (MH), inequitable gender attitudes, and childhood exposure to violence (e.g., Abramsky et al., 2011; Ramsoomar et al., 2021, 2023).

## MH, Substance Use Problems, and IPV Perpetration

MH problems in general and substance use (SU; drug and alcohol use) in particular are two prevalent problems among IPV perpetrators (Henrichs et al., 2015). Literature has shown that over a fifth of PIPs participants show evidence of an Axis I psychiatric disorder (Gondolf, 1999). Henrichs et al. (2015) compared criminological, psychopathological, and victimological profiles between a sample of IPV perpetrators with a sample of non-IPV perpetrators and concluded the same: men who enter PIPs have more psychopathology than nonviolent men, encompassing posttraumatic stress disorder (PTSD), depression, bipolar disorder, anxiety, intermittent explosive disorder, paranoia, and borderline PD symptoms (Henrichs et al., 2015). In relation to SU, in a sample of 1,290 male and 294 female IPV perpetrators who completed a court-mandated substance abuse evaluation, 41.4% of participants received alcohol, 36.0% received cannabis, 51.6% received cocaine, and 33.3% received an opioid use diagnosis (Crane et al., 2014).

Moreover, several studies have reported an increased risk of IPV perpetration among individuals with MH problems, including depression, anxiety disorders, panic disorders, SU disorders, and personality disorders, specifically antisocial personality disorder and borderline personality disorder (Crane et al., 2016; González et al., 2016; Oram et al., 2014; Okuda et al., 2015; Petersson et al., 2019; Spencer et al., 2022; Yu et al., 2019). Crane et al. (2014) examined symptomatology among a sample of 190 IPV perpetrators. Results indicated that participants diagnosed with bipolar and PTSD were more likely to perpetrate IPV than matched comparison. Moreover, MH problems (e.g., SU, personality disorders) can increase both the frequency and severity of violence (Cafferky et al., 2018; Collison & Lynam, 2021; Jackson et al., 2015). For example, O’Farrell et al. (2003) assessed occurrences of partner violence in the year preceding treatment for alcohol abuse among male patients, contrasting the findings with a non-alcohol abuser comparison group. Before treatment, 56% of patients with alcohol abuse exhibited violence toward their female partners, a rate four times higher than that observed in the comparison sample (14%). In addition, individuals who perpetrated IPV and had MH issues posed several challenges in treatment. As such, MH problems are strongly correlated with low treatment adherence, dropout, and recidivism (Hilton & Radatz, 2021; Romero-Martínez et al., 2019). For instance, IPV perpetrators with higher levels of psychopathology have an increased likelihood of dropping out of programs and reoffending (Carbajosa et al., 2017; Llor-Esteban et al., 2016).

Literature indicates that intervention programs for IPV perpetrators should be customized to address specific risk factors associated with these groups (Easton & Crane, 2016). Studies have demonstrated promising outcomes when intervention programs include treatments for MH issues (Karakurt et al., 2019; Nesset et al., 2019). Therefore, studies indicate that effectively decreasing MH issues is linked to a decrease in IPV perpetration (Cafferky et al., 2018). In recent years, several systematic reviews (e.g., Gannon et al., 2019; Pinto e Silva et al., 2023) have been conducted focusing on the effectiveness of interventions, but few have focused on IPV perpetrators with MH and SU problems. To our knowledge, two systematic reviews focused on this (e.g., Karakurt et al., 2019; Stephens-Lewis et al., 2021). In a 2019 systematic review and meta-analysis, Karakurt et al. (2019) evaluated the effectiveness of PIPs for male perpetrators of IPV, including only controlled experimental studies. They discovered that interventions that included substance abuse components were more effective compared to those without this component (Karakurt et al., 2019). Two years later, Stephens-Lewis et al. (2021) focused on intervention problems for IPV perpetrators with SU problems, including only randomized controlled trials or non-randomized controlled trials. The nine studies demonstrated some reductions in SU and IPV outcomes in the short term, but the results of the meta-analysis did not find significant differences between integrated interventions and their SU Treatment-As-Usual groups.

## Current Study

In this systematic review, we used an inclusive methodological approach. So, based on different research designs, we aimed to analyze the effectiveness of interventions targeting SU and MH problems in interventions with IPV perpetrators and their potential impact on perpetrators’ behavior and attitudes. More specifically, it aims to describe and assess the effectiveness of psychological interventions for IPV perpetrators with MH and SU problems. This systematic review extends previous ones (e.g., Karakurt et al., 2019; Stephens-Lewis et al., 2021; Travers et al., 2021) by incorporating MH problems alongside SU issues. Additionally, it uniquely includes justice-involved individuals, encompassing both men and women, which previous reviews did not address. Furthermore, this review does not impose limitations on the type of study design or the nature of the control groups, allowing for a more comprehensive and inclusive analysis.

## Methods

### Protocol and Registration

This systematic review followed the Preferred Reporting Items for Systematic Reviews and Meta-Analysis (PRISMA) guidelines (Moher et al., 2010). Moreover, the systematic review protocol was registered on OSF REGISTRIES (reference: 10.17605/OSF.IO/NUY69).

### Eligibility Criteria

PICOS schemes were used to define the inclusion criteria: Participants (P), Interventions (I), Comparisons (C), Outcomes (O), and Study Designs (S) (Thomas et al., 2021). Studies meeting the following criteria were considered for inclusion: (a) adult men and women perpetrators of IPV with SU disorders and/or MH problems (P); (b) those who underwent a rehabilitation program (I), and (c) having or not a comparison group (C). Moreover, the considered outcome was the effectiveness of psychological intervention, assessed through clinical changes and/or recidivism rates (O). All types of study designs were taken into consideration for this review (S). Finally, research papers written in English, Portuguese, or Spanish were included in this review. The exclusion criteria included: (a) books; (b) conference presentations; (c) systematic reviews and meta-analyses; and (d) papers not peer-reviewed (i.e., gray literature). No restrictions regarding the year of publication were made.

### Information Sources and Search Process

The subsequent equation was employed to ascertain the pertinent articles: (“marital offen*” OR “perpetrator” OR “spouse abus*” OR “batterer”) AND (intervention OR program* OR rehab* OR treatment OR therapy) AND (“alcohol abuse” OR “alcohol dependenc*” OR “alcohol problem” OR “substance abuse” OR “drug abuse” OR “drug dependenc*” OR “drug problem” OR “substance problem” OR “mental disorder” OR “mental problem” OR “mental health” OR “mental illness” OR “psychiatric illness” OR “psychiatric disorder”). In December 2023, a search utilizing the equation was run into five electronic databases searching by title, abstract, and keywords: B-On; Pubmed (Medline); PsycInfo; Science Direct; and Scopus. Moreover, the reference lists of several review articles focused on IPV perpetrators (Eckhardt et al., 2013; Expósito-Álvarez et al., 2023; Idriss-Wheeler et al., 2021; Karakurt et al., 2019; Nesset et al., 2019; Satyen et al., 2022; Stephens-Lewis et al., 2021; Stover et al., 2009; Timko et al., 2012; Travers et al., 2021) were examined to identify relevant manuscripts not identified through the database search.

### Study Selection

Studies identified through the equation-based search were imported into Rayyan software (Ouzzani et al., 2016), and duplicate entries were removed. Subsequently, two reviewers independently examined the titles and abstracts, and articles were chosen for full-text analysis based on whether they met the inclusion criteria. Any discrepancies were resolved through discussion.

### Data Extraction

Two reviewers independently extracted data from the selected studies using a codebook developed for this purpose. Data extraction process encompassed information on reference details (e.g., authors and year); studies’ characteristics (e.g., location); samples’ characteristics (e.g., size, age, sex, and ethnicity/race); design’s characteristics (e.g., design type and length of follow-up); intervention’s characteristics (e.g., setting, modality, number of sessions or hours, and complementary intervention); measurement characteristics (e.g., assessment measures and assessment of recidivism); intervention’s results (e.g., dropout/completion rate and efficacy). The process was carried out by the two authors and discussed with a senior author. Disagreements were discussed until a consensus was reached.

### Quality Assessment

The Mixed Methods Appraisal Tool (MMAT; Hong et al., 2018) was employed to assess the methodological quality of all included studies. The tool incorporates various items to assess the methodological quality, tailored to the specific characteristics of empirical studies (i.e., qualitative research, randomized controlled trials, non-randomized studies, quantitative descriptive studies, and mixed methods studies). Each of the criteria is classified as “yes,” “no,” or “don’t know.” Additionally, two authors independently evaluated the methodological quality of the studies. Any disagreements were resolved through discussions involving a third author.

### Data Analysis

The methodology employed involved a narrative synthesis approach, wherein narrative text and tables were utilized to succinctly summarize the gathered data. This framework empowers readers to evaluate outcomes considering discrepancies in study designs and potential sources of bias across the reviewed studies.

## Results

### Screening and Selection of Studies

**Figure 1.**
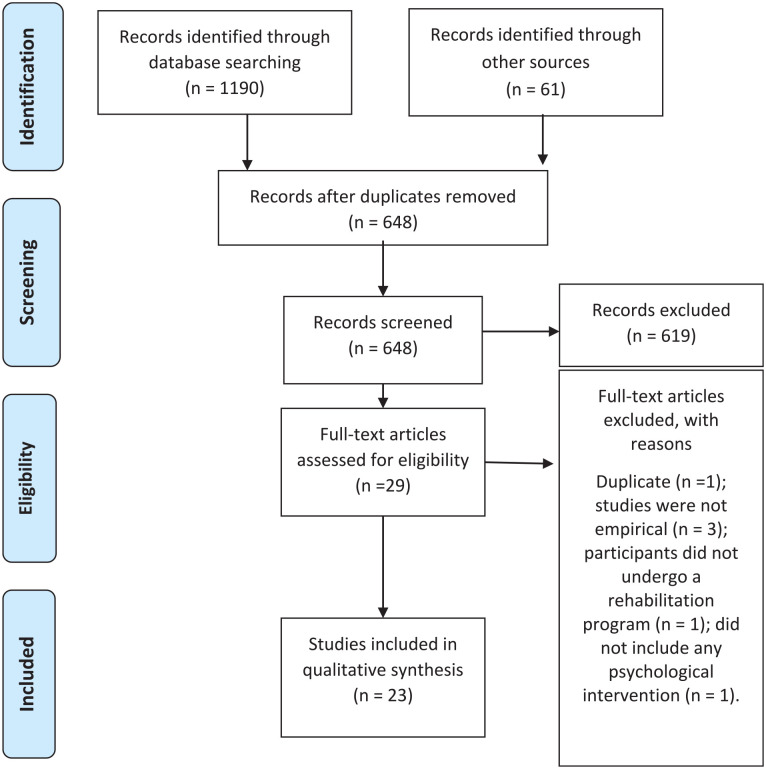
PRISMA flow diagram of the study selection process. PRISMA, Preferred Reporting Items for Systematic Reviews and Meta-Analysis.

In total, 1,190 articles were retrieved from the data searches, and 61 other studies were found through supplementary searches. After removing duplicates, 648 titles and abstracts remained and were screened for relevance. A total of 29 potentially relevant studies were selected for further examination. Then, one was found to be a duplicate, and five further studies did not meet the inclusion criteria. The main reasons for exclusion were: studies were not empirical (*n* = 3), participants did not undergo a rehabilitation program (*n* = 1) and did not include any psychological intervention (*n* = 1). Twenty-three were included in the final review. Figure 1 provides the PRISMA flow diagram illustrating the number of included studies in each selection process and the reasons for the studies’ exclusion.

### Quality Assessment

Among the included articles, most were designed as randomized control trials (RCT; *n* = 14). The remaining conducted a quantitative descriptive study (*n* = 5), a quantitative non-randomized study (*n* = 3), and one conducted a mixed method study (see Table 1).

**Table 1.** Study’s Sample and Intervention Characteristics.

Author, year	Country, setting recruited population	Design	Sample size (experimental groups vs. control group), gender	Treatment program	Intervention models, format, and timing	Intervention targets	Control
Brannen and Rubin (1996)	USA, community	Mixed methods	48 heterosexual couples, 22 vs. 26	CI	CBT, Group sessions	Nonspecific	GSI
Catalá-Miñana et al. (2013)	Spain, community	Descriptive	166 men	Contexto Program	CBT, Group sessions; 9 months	Nonspecific	N/A
Easton, Mandel, Babuscio, et al. (2007)	USA, SU outpatient facility	RCT	75 men, 38 vs. 37	SADV	CBT, Group sessions; 12 weeks, 90-min sessions (18 hr total)	Alcohol + drug abuse	TSF
Easton, Mandel, Hunkele, et al. (2007)	USA, SU outpatient facility	RCT	75 men, 38 vs. 37	SADV	CBT, Group sessions; 12 weeks, 90-min sessions (18 hr total)	Alcohol + drug abuse	TSF
Easton et al. (2018)	USA, SU outpatient facility	RCT	63 men, 29 vs. 34	SADV	CBT, individual sessions; 12 weeks	Alcohol + drug abuse	DC
Fals-Stewart et al. (2002)	USA, outpatient facility	RCT	80 heterosexual couples	BCT	CBT, individual and group sessions; 56 sessions	Alcohol + drug abuse	IBT
Gilchrist et al. (2021)	UK, SU outpatient facility	RCT	104 men, 54 vs. 50	ADVANCE + SU + TAU	Motivational, cognitive and dialectical behavioral therapies, 2–4 individual sessions; 12 group sessions	Alcohol + drug abuse	TAU
Gondolf (2009)	USA, community	Descriptive	479 men	N/A	CBT, individual and group sessions; 16 sessions	psychopathology	NT
Kraanen et al. (2013)	Netherlands, SU outpatient facility	RCT	52, 27 vs. 25 36 (69.2%) men	I-StoP	Motivational and CBT, individual sessions; 16 sessions	Alcohol + drug abuse	CBT-SUD+
Lila et al. (2016)	Spain, community	Descriptive	107 men	Contexto program	CBT, Group sessions; 32 sessions	Nonspecific	N/A
Lila et al. (2020)	Spain, community	Descriptive	286 men	Contexto program	CBT, Group sessions; 30–32 sessions	Nonspecific	N/A
Mbilinyi et al. (2011)	USA, community	RCT	124 men, 58 vs. 66	MET	MI, individual sessions; 1–3 sessions	Nonspecific	TAU
Murphy et al. (2018)	USA, community	RCT	228 men, 110 vs. 118	MET	MI, individual sessions; 4 sessions	Alcohol abuse	AE
Nesset et al. (2021)	Norway, outpatient facility	RCT	125 men, 67 vs. 58	CBT	CBT, individual and group sessions; 30 hr	Psychopathology	MBT
O’Farrell and Murphy (1995)	USA, outpatient facility	Quantitative non-randomized	176 heterosexual couples	BMT	BT, Group sessions; 6–8 pregroup sessions + 10 BMT sessions	Alcohol abuse	N/A
O’Farrell et al. (1999)	USA, outpatient facility	Quantitative non-randomized	75 heterosexual couples	BMT	BT, Group sessions; 6–8 pregroup sessions + 10 BMT sessions	Alcohol abuse	N/A
O’Farrell et al. (2004)	USA, outpatient facility	Quantitative non-randomized	303 heterosexual couples	BCT	CBT, Group sessions; 10–12 pregroup sessions; 10 BCT sessions	Alcohol abuse	N/A
Romero-Martínez et al. (2016)	Spain, community	Descriptive	116 men, 55 vs. 61	Contexto program	CBT, Group sessions; 30 sessions	Nonspecific	N/A
Satyanarayana et al. (2016)	India, SU inpatient facility	RCT	177 men, 88 vs. 89	ICBI	CBT, 8 sessions	Alcohol abuse	TAU
Schumacher et al. (2011)	USA, outpatient facility	RCT	Study 2: 23 men	MET	MI, 1 session	Alcohol abuse	N/A
Stover (2015)	USA, community	RCT	18 men	Fathers for change	Attachment, family systems, CBT, individual and group sessions; 4 months	Alcohol + drug abuse	IDC
Stuart et al. (2013)	USA, community	RCT	252 men, 123 vs. 129	SBP + BAI	CBT and MI, individual and group sessions; 40 hr + 90-min alcohol intervention	Alcohol abuse	SBP
Stuart et al. (2016)	USA, community	RCT	252 men, 123 vs. 129	SBP + BAI	CBT and MI, individual and group sessions; 40 hr + 90-min alcohol intervention	Alcohol abuse	SBP

*Note*. AE = alcohol education; BCT = behavioral couples therapy; BMT = behavioral marital therapy; BT = behavioral therapy; CBT = cognitive behavioral therapy; CBT-SUD+ = cognitive behavioral treatment addressing substance use disorders including only one session addressing partner violence; CI = Couples Intervention; DC = drug counseling; GSI = gender-specific intervention; IBT = individual-based treatment; ICBI = integrated cognitive-behavioral intervention; IDC = individual drug counseling; I-StoP = integrated treatment for substance abuse and partner violence; MBT = mindfulness-based therapy; MET = motivational enhancement therapy; MI = Motivational interview; N/A = not applicable; NT = not therapy; RCT = randomized control trial; SADV = substance abuse domestic violence; SBP + BAI = SBP plus an alcohol intervention; SBP = Standard Batterer Program; SU = substance use; TAU = treatment as usual; TSF = Twelve Step Facilitation.

Of the 23 studies, 3 showed all the criteria of excellent (Easton, Mandel, Hunkele, et al., 2007; Easton et al., 2018; Satyanarayana et al., 2016), 4 presented 4 out of 5 criteria of excellent (Brannen & Rubin, 1996; Mbilinyi et al., 2011; Murphy et al., 2018; Stuart et al., 2013), 7 showed 3 out of 5 criteria (Catalá-Miñana et al., 2013; Fals-Stewart et al., 2002; Nesset et al., 2021; O’Farrell & Murphy, 1995; O’Farrell et al., 1999; Schumacher et al., 2011; Stover et al., 2015), 7 presented 2 out of 5 criteria (Gilchrist et al., 2021; Gondolf, 2009; Kraanen et al., 2013; Lila et al., 2016, 2020; O’Farrell et al., 2004; Romero-Martínez et al., 2016; Stuart et al., 2016), and 1 showed 1 out of 5 criteria (Easton, Mandel, Babuscio, et al., 2007). No articles were excluded based on their quality assessment; however, information about the main results of the studies and their level of bias in conducting the research is presented in the table.

### Characteristics of Included Studies

#### Reference Information, and Study’s and Sample Characteristics

Table 1 includes a summary of the main characteristics of the included studies. The year of publication of the articles varied between 1995 (O’Farrell & Murphy, 1995) and 2021 (Gilchrist et al., 2021; Nesset et al., 2021). The year with the highest number of publications was 2016 (*n* = 4), followed by 2013 (*n* = 3), 2007 (*n* = 2), 2011 (*n* = 2), 2018 (*n* = 2), and 2021 (*n* = 2). Most of the studies included were from USA (*n* = 15; Brannen & Rubin, 1996; Easton, Mandel, Babuscio, et al., 2007; Easton, Mandel, Hunkele, et al., 2007; Easton et al., 2018; Fals-Stewart et al., 2002; Gondolf, 2009; Mbilinyi et al., 2011; Murphy et al., 2018; O’Farrell & Murphy, 1995; O’Farrell et al., 1999; O’Farrell et al., 2004; Schumacher et al., 2011; Stover, 2015; Stuart et al., 2013, 2016). The remaining were from Spain (*n* = 4; Catalá-Miñana et al., 2013; Lila et al., 2016, 2020; Romero-Martínez et al., 2016), Norway (*n* = 1; Nesset et al., 2021), India (*n* = 1; Satyanarayana et al., 2016), UK (*n* = 1; Gilchrist et al., 2021), and Netherlands (*n* = 1; Kraanen et al., 2013).

The sample size of the studies ranged between 23 (Schumacher et al., 2011) to 303 (O’Farrell et al., 2004) perpetrators of IPV. The mean age of the participants ranged between 30.19 (Stover, 2015) and 43.50 years (O’Farrell & Murphy, 1995). From the studies, it was possible to observe that all samples were entirely composed of male individuals (*n* = 17), except for five studies which included couples but the perpetrator of IPV was the man (Brannen & Rubin, 1996; Fals-Stewart et al., 2002; O’Farrell & Murphy, 1995; O’Farrell et al., 1999; O’Farrell et al., 2004), and one study, which predominantly had a male sample (Kraanen et al., 2013) (see Table 1).

#### Characteristics of the Intervention Programs

The studies included examined 13 treatment programs (K). The intervention programs implemented were the Substance Abuse Domestic Violence (SADV) Program (Easton, Mandel, Babuscio, et al., 2007; Easton, Mandel, Hunkele, et al., 2007; Easton et al., 2018), the Behavioral Couples Therapy (BCT) Program (Fals-Stewart et al., 2002; O’Farrell et al., 2004), the ADVANCE (Gilchrist et al., 2021), the Integrated Treatment for Substance abuse and Partner (I-StoP) Violence (Kraanen et al., 2013), the Motivational Enhancement Therapy (MET; Mbilinyi et al., 2011; Murphy et al., 2018; Schumacher et al., 2011), the CBT Program (Nesset et al., 2021), the Behavioral Marital Therapy (BMT) Program (O’Farrell & Murphy, 1995; O’Farrell et al., 1999), the Father for Change Program (Stover, 2015), a Standard Batterer Program plus and Alcohol Intervention (SBP + BAI; Stuart et al., 2013, 2016), the Integrated Cognitive-Behavioral Intervention (ICBT; Satyanarayana et al., 2016), the Contexto Program (Catalá-Miñana et al., 2013; Lila et al., 2016, 2020; Romero-Martínez et al., 2016), and a Couples Intervention (Brannen & Rubin, 1996). The remaining intervention program did not identify a specific name for the treatment program (Gondolf, 2009). Seven interventions were based on CBT; 3 interventions were a combination of CBT with other theories, including Motivational Interviewing (MI) techniques; and the remaining used only MI techniques (*K* = 1), Behavioral Therapy (*K* = 1), and a combination of Motivational, Cognitive and Dialectical Behavioral Therapy (*k* = 1).

Most of the intervention programs were specific interventions for alcohol and/or drug abuse and MH issues in IPV perpetrators, and three were nonspecific for this phenomenon (MET; Contexto Program; CI). None of the intervention programs specifies whether they adhere to the principles of the RNR model.

Ten studies included intervention programs delivered in group formats, seven had individual and group sessions, and four included individual sessions. Moreover, two studies did not specify the sessions’ format (Satyanarayana et al., 2016; Schumacher et al., 2011). Eleven studies included participants in outpatient facilities; 11 studies included participants in the community; and the remaining in inpatient facilities (*n* = 1). However, participants were referred to the intervention by the court (*n* = 11) or invited to participate because they were in treatment clinics (*n* = 10). Two studies included participants referred to the intervention by the court and referenced by healthcare professionals (Gondolf, 2009; Satyanarayana et al., 2016).

### Main Findings of the Analyzed Studies

Intervention outcomes are presented according to the treatment aim and specialization of treatment: specific intervention integrated IPV and SU, nonspecific intervention for SU among IPV perpetrators, and specific intervention for IPV perpetrators with MH. Specialized programs are programs aimed at IPV and/or SU. Table 2 presents the main findings of the studies along with their quality.

**Table 2.** Main Outcomes.

Author, year	MMAT	Dropout rates	Recidivism definition	Main outcomes/dropout rates
Brannen and Rubin (1996)	4/5	n/a	Self-report of further contact with either the police or court.	- Participants with alcohol abuse had better outcomes with a couple’s approach. - However, the assigned intervention did not significantly affect psychological abuse, communication, or marital satisfaction after controlling for alcohol consumption. Recidivism rates: 7.1%–8.3%
Catalá-Miñana et al. (2013)	3/5	Alcohol problems condition: 31.4% Non-alcohol problems condition: 16.3%	n/a	- High dropout rate in the intervention for those with alcohol problems. - Participation significantly reduced alcohol consumption. Treatment participation brings positive changes in the risk of recidivism, attitudes toward violence, depression symptoms, and social support, regardless of alcohol use. No post-treatment changes were observed in self-esteem, responsibility attribution, perception of intimate support, community integration, and support from formal systems.
Easton, Mandel, Babuscio, et al. (2007)	1/5	n/a	n/a	- Participants with alcohol + drug issues attended fewer sessions, had fewer abstinent days from alcohol, and had more positive breathalyzer results than those with only alcohol problems. - Participants with alcohol + drug issues showed significantly more impairments in anger management styles from pre- to post-treatment. No significant differences between groups in verbal and physical aggression.
Easton, Mandel, Hunkele, et al. (2007)	5/5	17%	n/a	- No significant differences in session attendance between the two treatments. Participants in the experimental condition reported significantly fewer days of alcohol use and a trend toward achieving a greater reduction in the frequency of violent episodes over time compared to the control condition.
Easton et al. (2018)	5/5	IC = 31.03%; CC = 26.47%	n/a	- No significant differences in session attendance between the two treatments. In the experimental condition, participants had fewer cocaine-positive toxicology screens and breathalyzer results during treatment. They were also less likely to engage in aggressive behavior proximal to a drinking episode and reported fewer episodes of violence than the control group.
Fals-Stewart et al. (2002)	3/5	n/a	n/a	- In the year following the intervention, fewer couples in the experimental condition reported male-to-female physical aggression than in the control condition. For participants in the experimental condition, most outcomes (e.g., percent days of alcohol and drug abuse, percent days of drug use, percent days of heavy alcohol use) were more positive.
Gilchrist et al. (2021)	2/5	n/a	n/a	Participants in the experimental condition showed improvements in almost all scales (e.g., mental health, IPV in the past 4 months, self-management).
Gondolf (2009)	2/5	n/a	Re-assault refers to physical abuse reported by the individual’s female partner during phone interviews, while re-arrests are records from a state-wide criminal history database.	- Participants receiving mental health treatment significantly increased batterer program completion. - Participants receiving mental health treatment were one-third less likely to re-assault, but the difference is not statistically significant.
Kraanen et al. (2013)	2/5	IC = 56%; CC = 66.7%	n/a	- Participants in both conditions significantly improved regarding SU, IPV perpetration, and psychopathology symptoms at post-treatment compared with pretreatment, but there were no differences between conditions. - Participants in both conditions did not improve in marital satisfaction. Dropout rates did not differ significantly between conditions.
Lila et al. (2016)	2/5	30.84%	n/a	- Participants with alcohol problems did not present higher dropout rates than participants without alcohol problems but presented a reduction in alcohol consumption. - Regardless of the alcohol use, participants showed improvements in all outcome variables (i.e., risk of recidivism, attributions, and attitudes toward violence, depression symptoms, and social support). There are no significant differences in recidivism rates among participants with or without alcohol problems who completed the intervention.
Lila et al. (2020)	2/5	Alcohol problems condition: 36%; Non-alcohol problems condition: 22.67%	Additional incidents of IPV, other offenses within intimate partner contexts, or any violations of court-mandated conditions.	- Participants with alcohol problems presented higher dropout rates but showed a reduction in alcohol abuse. - Regardless of alcohol abuse problems, perpetrators who completed the program showed improvements in all intervention outcomes analyzed (i.e., risk of recidivism, attributions, and attitudes toward violence, depression symptoms, and social support). There are no significant differences in recidivism rates among participants with (10.96%) or without alcohol problems (8.23%) who completed the intervention.
Mbilinyi et al. (2011)	4/5	IC = 13.37%; CC = 0%	n/a	- Participants in treatment conditions showed a reduction in IPV behavior, increasing motivation for treatment seeking, and changing perceived norms for IPV and SU than control groups. There are no differences between groups in SU.
Murphy et al. (2018)	4/5	IC = 10%; CC = 14.4%	n/a	Participants showed improvements in alcohol abstinence, heavy drinking, illicit drug use, and partner violence regardless of the treatment condition.
Nesset et al. (2021)	3/5	IC = 14.5%; CC = 15.1%	n/a	- Participants showed improvements in anxiety and depression symptoms as well as in difficulties in emotion regulation regardless of the treatment condition. In the 12-month follow-up, the total symptom scores remained high in both groups.
O’Farrell and Murphy (1995)	3/5	n/a	n/a	- Violence decreased significantly in prevalence and frequency in the year after intervention but remained significantly elevated relative to the matched controls. - After intervention, remitted alcoholics presented better results in marital violence levels than relapsed alcoholics.
O’Farrell et al. (1999)	3/5	15%	n/a	- Violence was significantly reduced after the intervention. After intervention, remitted alcoholics presented better results in marital violence levels than relapsed alcoholics.
O’Farrell et al. (2004)	2/5	12.10%	n/a	- Violence decreased significantly from the year before intervention. Significant violence reductions occurred for patients whose alcoholism was remitted after the intervention.
Romero-Martínez et al. (2016)	2/5	n/a	n/a	- Participants improved in cognitive empathy and cognitive flexibility, with participants with lower alcohol consumption showing a higher improvement in these skills than participants with higher alcohol consumption.
Satyanarayana et al. (2016)	5/5	n/a	n/a	- Participants in the treatment condition reported significantly lower IPV perpetration than participants in the control group. Alcohol consumption in the men was not significantly different between the groups.
Schumacher et al. (2011)	3/5	n/a	n/a	- Participants in treatment conditions reported better results in help-seeking, leading to marginally significant enhancements in motivation and self-reported intimacy than others. At follow-up, both groups showed improvements in self-reported alcohol outcomes, anger, and verbal and physical aggression.
Stover (2015)	3/5	IC = 33%; CC = 67%	n/a	Participants in treatment conditions were more likely to complete treatment, reported significantly greater satisfaction with the program, and reported a trend toward less IPV than the control group.
Stuart et al. (2013)	4/5	n/a	Arrest records for domestic violence charges, domestic assaults, or restraining orders filed against participants within 12 months after their baseline assessment.	- Participants in the treatment condition reported significant reductions in drinks per drinking day at three months but not 6 or 12-month follow-ups than those in the control group. - Participants in treatment condition reported significantly greater abstinence at 3- and 6-month follow-up. - No significant differences in physical IPV between conditions. 13.8% vs. 13.1% arrested rates of experimental and control, respectively.
Stuart et al. (2016)	2/5	n/a	n/a	Participants in treatment condition had greater days abstinent, less physical violence perpetration, and fewer injuries to partners than control.

*Note*. CC = control condition; IC = intervention condition; IPV = intimate partner violence; MMAT = Mixed Methods Appraisal Tool; SU = substance use.

#### Specific Intervention Integrated IPV and SU

SADV is a CBT approach that addresses SU, interpersonal violence, and the interplay between the two and was evaluated in three studies (Easton, Mandel, Babuscio, et al., 2007; Easton, Mandel, Hunkele, et al., 2007; Easton et al., 2018). The first study analyzed the program’s effectiveness by comparing it with an approach focused on IPV but not specific to substance problems (Easton, Mandel, Hunkele, et al., 2007). Another study evaluated its effectiveness by comparing it with the Twelve Step Facilitation (TSF) program but for participants with and without drug use during treatment (Easton, Mandel, Babuscio, et al., 2007). Finally, the program’s effectiveness was analyzed compared to an equally intensive individual therapy intervention targeting SU (drug counseling) (Easton et al., 2018). The studies concluded there are no differences between SADV and the other two intervention programs in the number of sessions attended by the participants (Easton, Mandel, Babuscio, et al., 2007; Easton, Mandel, Hunkele, et al., 2007; Easton et al., 2018). When compared with TSF, SADV revealed significantly more positive changes in the number of days abstinent from alcohol during the 12 weeks of treatment and physical violence. However, there was no significant difference between treatments in the number of days abstinent from alcohol use or the frequency of physical violence at the follow-up (Easton, Mandel, Babuscio, et al., 2007). Moreover, the effects of SADV are more positive when participants had only alcohol problems compared to those with both alcohol and drug issues (Easton, Mandel, Babuscio, et al., 2007). When compared with DC, SADV produces reductions in the number of cocaine and breathalyzer-positive tests during treatment, in the occurrences of aggression on days of drinking, and in the days that participants perpetrated IPV in follow-up. However, it is important to note that while these reductions were observed, statistical analyses did not reveal significant differences between SADV and DC in total, physical, and verbal aggression (Easton et al., 2018).

The BCT, BMT, and SBP + BAI are specific programs aimed at IPV perpetrators with SU problems and have been evaluated in two studies each. However, unlike the previous program, the primary targets were not IPV. Any changes in IPV should be considered as a positive additional benefit or “side effect” of the focus on SU. The BCT program comprises sessions focusing on drug abuse counseling and teaching skills to enhance partner relationship adjustment, utilizing principles of CBT. Similarly, BMT aims to cultivate support for abstinence and enhance relationship functioning through behavioral therapy techniques. Lastly, the SBP + IPV BAI incorporates a standard batterer intervention program along with a single 90-min motivational session addressing alcohol-related issues (Stuart et al., 2013, 2016). The results revealed that these programs produce more positive outcomes than the comparison intervention, particularly in terms of reducing the level of physical aggression displayed (Fals-Stewart et al., 2002). Furthermore, when compared to a control group without substance problems, violence, although reduced, remained high, and the pattern of consumption influenced the results (O’Farrell & Murphy, 1995; O’Farrell et al., 1999; O’Farrell et al., 2004). Participants in the remitted condition showed better results than those in the relapse condition (O’Farrell & Murphy, 1995; O’Farrell et al., 1999). When analyzing recidivism rates, the programs produce significant reductions (Stuart et al., 2013).

The other programs appear in only one study each (i.e., ADVANCE, I-StoP, Fathers for Change, and ICBT). The intervention programs produced improvements in various selected outcomes such as self-management, physical and verbal IPV, MH, and alcohol consumption between pre- and post-tests. However, there are some discrepancies in terms of statistical significance. Some targeted outcomes have shown significant differences between the intervention and comparison groups (Gilchrist et al., 2021; Satyanarayana et al., 2016; Stover, 2015). Notably, improvements were observed in certain areas, while others, despite positive evolution from pre- to post-test, did not exhibit differences between conditions (Kraanen et al., 2013; Satyanarayana et al., 2016). For instance, Satyanarayana et al. (2016) found a reduction in consumption patterns without distinctions between the experimental and control groups. However, a more substantial reduction in terms of IPV severity was noted in the experimental group (see Table 2).

#### Nonspecific Interventions for IPV with SU

Four studies evaluated the effectiveness of the Contexto program with perpetrators of IPV with alcohol problems (Catalá-Miñana et al., 2013; Lila et al., 2016, 2020; Romero-Martínez et al., 2016). Two studies used clinical change as the outcome (Catalá-Miñana et al., 2013; Romero-Martínez et al., 2016), while the other two incorporated two outcomes, including clinical change and recidivism rates (Lila et al., 2016, 2020). The program is a community-based intervention program grounded in the ecological model framework. It consists of three phases (evaluation, intervention, and follow-up), with the intervention phase being based on CBT. The results showed that the intervention program led to a reduction in alcohol consumption (Catalá-Miñana et al., 2013; Lila et al., 2016, 2020). Moreover, intervention participation led to the reduction of the risk of recidivism, depressive symptoms, attributions of responsibility, attitudes toward violence, and sexism and improvements in social integration regardless of alcohol abuse problems (Catalá-Miñana et al., 2013; Lila et al., 2016, 2020). Romero-Martínez et al. (2016) also analyzed the effectiveness of the program in promoting cognitive flexibility and empathy in participants with different levels of alcohol consumption. They concluded that, in the case of high consumption patterns, participants obtained lower scores in the Eyes Test, perspective-taking, and cognitive flexibility compared to those with lower consumption levels (Romero-Martínez et al., 2016). Lastly, the studies concluded that there were no differences in recidivism after participating in the program in the groups with or without consumption (Lila et al., 2016, 2020). However, results are mixed concerning dropout rates: two studies showed that men with alcohol problems dropped out of treatment more often (Catalá-Miñana et al., 2013; Lila et al., 2020), while in another there were no significant differences between the groups (Lila et al., 2016).

MET program was analyzed in three studies, with significant variations in the number of sessions (Mbilinyi et al., 2011: 1–3 sessions; Murphy et al., 2018: 4 sessions; Schumacher et al., 2011: 1 session). Analyzing effectiveness through clinical change, it was concluded that there are outcomes that improved over time but did not differ between conditions (i.e., alcohol consumption, violence; Mbilinyi et al., 2011; Murphy et al., 2018; Schumacher et al., 2011) except for a few variables (i.e., perceived norms, perceived drink norms, increasing motivation) (Mbilinyi et al., 2011; Schumacher et al., 2011).

Lastly, CI was evaluated in one study (Brannen & Rubin, 1996). The intervention utilizes a CBT approach, employing a core curriculum consistent with standard batterer intervention practices. The study compared the effectiveness of this approach with the gender-specific group intervention across various measures, including conflict resolution ability, levels of violence, communication within the dyadic relationship, marital satisfaction, and recidivism prevention (Brannen & Rubin, 1996). For many variables, the differences between conditions were not significant, indicating that the specific condition did not significantly impact the outcome (e.g., psychopathology, level of communication, marital satisfaction). The rates of recidivism range between 7.1% and 8.3% for couples’ groups and gender-specific groups, respectively. However, among individuals with a history of alcohol abuse, the couples’ approach demonstrated clear superiority.

#### Specific Intervention for IPV and MH Issues

Two studies assess the effects of MH treatment on participants in PIPs who have MH issues (Gondolf, 2009; Nesset et al., 2021). These studies employ different outcome measures to evaluate the effectiveness of the treatment. Nesset et al. (2021) evaluated clinical change, and Gondolf (2009) the re-assault rates, program completion, and other indicators of abuse. Nesset et al. (2021) compared the effectiveness of cognitive-behavioral group therapy with mindfulness-based stress reduction group therapy. They concluded that anxiety, depression, and emotional regulation difficulties decreased in experimental and control groups at both time points. Still, the total symptoms remained high at the 1-year follow-up. Similar results were obtained by Gondof (2009), who showed that there were no statistically significant differences in program completion for perpetrators of IPV who underwent supplementary treatment for MH problems. Additionally, they found that men who received MH treatment were one-third less likely to re-assault, although the differences were not statistically significant (no-treatment 30% vs. treatment 19%) (see Table 2).

## Discussion

The current systematic review aimed to analyze the effectiveness of interventions targeting SU and MH problems among IPV perpetrators and their potential impact on individuals’ behavior and attitudes. Our review provides additional insights and addresses specific gaps not fully explored in the previous works (e.g., Karakurt et al., 2019; Stephens-Lewis et al., 2021; Travers et al., 2021). Our systematic review extends the current understanding in several key areas: it specifically includes studies examining the efficacy of PIPs for individuals with MH problems, also incorporates studies focusing on justice-involved individuals, providing a unique perspective on how intervention programs operate within the criminal justice system, thereby shedding light on the specific needs and outcomes of this subgroup. Additionally, our review includes studies that examine both male and female perpetrators and all types of designs (i.e., RCT, non-RCT, quantitative descriptive, and mixed method). The search returned 23 relevant papers describing 13 intervention programs. These were further subdivided into 15 studies testing a specific treatment for SU among IPV perpetrators, 6 studies on nonspecific interventions for SU among IPV perpetrators, and 2 studies on specific interventions targeting MH among IPV perpetrators.

Most studies were conducted in the United States of America (USA), which is unsurprising given that PIPs were primarily developed and implemented in the USA, where they proliferated and became a popular punitive measure (Bowen, 2011). Indeed, this evidence has been corroborated in other reviews (e.g., Cunha, Pedrosa, et al., 2024; Pinto e Silva et al., 2023; Travers et al., 2021). Moreover, most intervention programs included were administered within community settings, even though participants were predominantly justice-involved individuals. This is surprising, as the literature has demonstrated that incarcerated IPV perpetrators exhibit a higher likelihood of SU, more severe personality disorders, and elevated scores on psychopathological symptoms compared to non-incarcerated IPV perpetrators, which may indicate a higher need to provide treatment in the prison system (e.g., Cunha & Gonçalves, 2018; Fernández-Montalvo et al., 2012; García-Jiménez et al., 2014). Beyond this, only one study included female perpetrators of IPV; however, it did not conduct statistical analysis based on gender (Kraanen et al., 2013). Furthermore, the program implemented is the same for both men and women, despite indications in the specialist literature that women have different criminogenic needs (Laskey, 2016).

In addition, most studies evaluate intervention programs based on CBT techniques. This trend suggests a move away from the adoption of “third-wave” therapies in addressing persons with IPV convictions within the field of MH (e.g., Zarling & Russell, 2022). The utilization of third-wave therapies has been advocated due to the suboptimal long-term outcomes associated with CBT (Johnsen & Friborg, 2015), with some promising results among justice-involved individuals (Cunha, Pereira, et al., 2024). However, four of these studies also incorporate motivation techniques either alongside CBT or in isolation. While based on a limited sample size of four studies, this outcome carries notable significance. The literature underscores the importance of addressing the motivation of IPV perpetrators, given their notably high dropout rates and the consequences of non-treatment completion (e.g., dropout is a predictor of IPV recurrence) (Cunha et al., 2023; Cunha, Pedrosa, et al., 2024; Lila et al., 2019). As a result, SU emerges as a predictive factor for discontinuing intervention programs (Cunha, Pedrosa, et al., 2024). Therefore, it is imperative not only to implement programs that specifically target SU but also to incorporate scientifically proven effective techniques, such as MI, to address this issue (Pinto e Silva et al., 2023; Soleymani et al., 2018).

Moreover, none of the intervention programs specify whether they adhere to the principles of the RNR model (Bonta & Andrews, 2016). This lack of specification can be problematic for several reasons. First, interventions have proven to be more effective when they meet these principles (e.g., Travers et al., 2021). Second, when complying with these principles, we assume that there was a psychological assessment of each perpetrator and that the group’s risk level is similar. The creation of heterogeneous therapeutic groups can compromise the effectiveness of the results, and the presence of varied psychological profiles, such as those seen in cases of psychopathy, can further disrupt group dynamics and functioning (Hilton & Ennis, 2020).

Overall, the current systematic review provides evidence of the effectiveness of specific and nonspecific interventions targeting SU in IPV perpetrators. Positive outcomes have been found in alcohol and drug reduction (e.g., Catalá-Miñana et al., 2013; Easton, Mandel, Babuscio, et al., 2007; Easton, Mandel, Hunkele, et al., 2007; Easton et al., 2018; Fals-Stewart et al., 2002), violence reduction and believes about IPV, SU, and violence (e.g., Easton, Mandel, Hunkele, et al, 2007; Fals-Stewart et al., 2002; Gilchrist et al., 2021; Kraanen et al., 2013), risk of recidivism reduction (e.g., Lila et al., 2016, 2020), and cognitive flexibility and empathy (Romero-Martínez et al., 2016). However, only seven of the included studies demonstrated a reduction in bias. These results must be considered alongside their inherent limitations; nevertheless, they offer valuable insights for the development of future programs, which should be evaluated through RCTs.

Results from our systematic review showed that only two intervention programs focused on MH in IPV perpetrators, without the intervention being enough to reduce the symptoms for non-clinical scores and to reduce recidivism in a significant way. At the same time, these are two of the studies that present low quality. This outcome concerns us, either due to the limited number of interventions and their outcomes. The literature has demonstrated that individuals perpetrating IPV exhibit a higher prevalence of psychopathological disorders compared to non-perpetrators (Henrichs et al., 2015). This factor also constitutes a risk factor for IPV’s perpetration, severity, and frequency (Cafferky et al., 2018; Collison & Lynam, 2021; Jackson et al., 2015). Furthermore, MH issues can undermine the effectiveness of programs designed to mitigate marital violence if they are not adequately addressed (Sesar et al., 2018). On the contrary, MH treatments for IPV perpetrators may yield additional indirect benefits in reducing IPV by impairing psychological and social skills. These skills include enhancing communication, stress, and anger management abilities, as well as diminishing social isolation. These factors collectively contribute to potentially lowering the incidence of IPV (Tol et al., 2019). The development and validation of intervention programs in these areas are extremely urgent. The results presented can provide critical support at this stage, along with the primary prevention programs that have shown positive results in some countries. A significant number of primary prevention interventions in these regions (i.e., Global South) are demonstrating reductions in IPV, alongside decreases in men’s alcohol abuse and improvements in MH. These interventions, designed to prevent violence before it starts, are often implemented in settings with a high prevalence of IPV (e.g., Doyle et al., 2023; Murray et al., 2020).

### Strengths and Limitations of This Review

This systematic review allows us to understand the potential benefits of including SU treatment in IPV perpetrators’ programs to reduce alcohol and drug consumption, diminish violence, and decrease recidivism rates regardless of the specificity of the programs. Besides, interventions that address MH issues are scarce and have limited positive outcomes.

Despite the contributions, some limitations should be noted. The first limitation identified was the poor quality of most studies and the high risk of bias in the conclusions presented. Second, there is a high prevalence of studies conducted in the USA compared to other countries, as well as the absence of studies in languages other than English and Spanish. This absence hinders a comprehensive understanding of interventions utilized in other countries to investigate the effectiveness of addressing SU and MH issues among IPV perpetrators. Third, the information provided by the studies on certain variables (e.g., intervention format and dropout rates) may have limited a more thorough understanding of this issue, as some studies did not specify all this information. Fourth, most of the study samples were composed of males, which did not allow for the generalization of results to women. Fifth, the wide variation in the designs used, the number of sessions held, the duration of each session, the format (individual vs. group), and assessment methods in determining IPV and SU behaviors hinder our ability to draw reliable conclusions regarding the effectiveness of such programs with IPV perpetrators. Sixth, the definition of recidivism varied across studies, which complicates comparisons between them. Recidivism is traditionally regarded as the optimal measure for assessing intervention program effectiveness; however, its utility is increasingly scrutinized due to the absence of a universally accepted definition and the presence of unreported cases that obscure accurate interpretation of results (Andersen & Skardhamar, 2017; Beaudry et al., 2021). Therefore, in addition to the clinical assessments that are becoming more common for evaluating program effectiveness (e.g., Cunha, Pedrosa, et al., 2024; Sousa et al., 2022, 2024), recidivism rates should incorporate legal records, sources of information from both perpetrators and victims and self-reports. Lastly, most of the studies included do not take into account the occurrence of bidirectional violence. Research has shown that bidirectional violence is a common pattern of violence, with both men and women can be victims and/or perpetrators (Machado et al., 2024). In this sense, it would be pertinent for future studies to assess the occurrence of this type of violence and whether the intervention takes this into account.

### Conclusion: Future Research

The main objective of this systematic review was to examine how interventions aimed at addressing SU and MH issues in IPV perpetrators impact their behavior and attitudes, assessing their effectiveness. This study enables us to establish the positive effects of SU interventions on alcohol and drug consumption, violence, and recidivism rates. However, it also shows us that the interventions that address MH problems in this population are scarce and have limited effects. Lastly, it also indicates that there is a scarcity of studies conducted outside the USA, particularly those implemented within the prison context and targeting female perpetrators. Still, the development of systematic reviews, meta-analyses, and other studies analyzing the effectiveness of SU and MH interventions in IPV perpetrators remains essential. Specifically, more RCTs are necessary to evaluate the effectiveness of interventions targeting SU and MH among IPV perpetrators with lower bias. Moreover, more research is needed to better understand the impact of these interventions on female IPV perpetrators, as well as their efficacy in prison settings and countries outside the USA (see Tables 3 and 4). In addition, it is imperative that future treatment studies incorporate the perspective of both partners, expanding the focus to include an examination of the partner’s behavior and the contextual factors surrounding the violence.

**Table 3.** Key Findings of the Systematic Review.

Key findings
Positive effects have been observed in SU interventions, including reductions in alcohol and drug consumption, instances of violence, and recidivism rates. These benefits are consistent across various types of interventions, regardless of their specificity.
The present systematic review concluded that there are few research studies analyzing the effectiveness of mental health interventions for IPV perpetrators.
The limited quality of most studies, coupled with the high risk of bias in their conclusions, constrained the overall conclusions drawn from the research.
Most of the study samples consisted of males, limiting the generalizability of results to women.

*Note*. IPV = intimate partner violence; SU = substance use.

**Table 4.** Implications for Research, Practice, and Policy.

Implications
More randomized clinical trials are necessary to evaluate the effectiveness of SU and MH interventions among IPV perpetrators.
More studies are needed to understand the impact of these interventions among the women population with IPV problems.
Further research should focus on prison settings and other countries beyond the USA.
More intervention programs are needed for persons who both have MH problems and engage in partner abuse.
SU and mental health problems among IPV perpetrators should be appropriately targeted in the intervention.

*Note*. IPV = intimate partner violence; MH = mental health; SU = substance use.
